# Gonorrhea and syphilis co-infection and related risk factors in HIV patients from Shiraz, South of Iran

**DOI:** 10.22088/cjim.9.4.397

**Published:** 2018

**Authors:** Farzaneh Ghassabi, Yalda Malekzadegan, Hadi Sedigh Ebrahim-Saraie, Hamid Heidari, Mozhgan Sabet, Abdollatif Bagheri, Narges Bagheri, Hadi Raeisi Shahraki, Alireza Hasanabadi, Mohammad Motamedifar

**Affiliations:** 1 Department of Bacteriology and Virology, School of Medicine, Shiraz University of Medical Sciences, Shiraz, Iran; 2 Shiraz HIV/AIDS Research Center, Institute of Health, Shiraz University of Medical Sciences, Shiraz, Iran; 3 Student Research Committee, Shiraz University of Medical Sciences, Shiraz, Iran; 4 Department of Biostatistics and Epidemiology, School of health, Shahrekord University of Medical Sciences, Shahrekord, Iran

**Keywords:** Sexually transmitted disease, Human immunodeficiency virus, Neisseria gonorrhea, Treponema pallidum

## Abstract

**Background:**

*Neisseria gonorrhea* and *Treponema pallidum* as an obligate human pathogen are two common causes of sexually transmitted diseases (STDs). The present study aimed to determine the prevalence of *N. gonorrhoeae *and *T. pallidum* among human immunodeficiency virus (HIV) patients in the southwest Iran.

**Methods:**

This retrospective study was performed from 2004 to 2013, on HIV patients who were tested for detection of gonorrhea and syphilis infection at Shiraz HIV/AIDS Research Center. ELISA technique was used for preliminary detection of HIV and confirmed by a Western Blotting test. Gonorrhea was routinely diagnosed using direct Gram-staining and culturing on selective agar. Syphilis was routinely diagnosed by RPR test.

**Results:**

Of the 806 HIV patients, 39 (2.6%) cases had co-infection with gonorrhea. Compared with mono-HIV infected patients, gonorrhea was significantly more likely among the males (69.4% vs. 92.3%, P=0.002). History of addiction and prison seems to be a significant risk factor for gonorrhea infection (P<0.05). Also, the mean of CD4+lymphocyte was higher among gonorrhea infected patients (368±238 vs. 415±328). Logistic regression analysis showed that sexual contact increased risk of gonococcal infection about 4 fold (OR: 4, CI: 1.7-9.39, P=0.001). None of the HIV patients had syphilis co-infection.

**Conclusions:**

As a preliminary survey, our findings provided unique information on the prevalence of gonorrhea and syphilis co-infections among HIV patients. Moreover, we introduced the main risk factors associated with gonorrhea co-infection in HIV patients in our region including gender, history of addiction and prison, CD4+lymphocyte count, and transmission routes for effective management of STDs.

Sexually transmitted diseases (STDs) are one the major public health concern in the world (1). *Neisseria gonorrhea* is an obligate human pathogen responsible for gonorrhea as one of the commonest sexually STDs (2). The World Health Organization (WHO) estimates a significant increase in the global incidence of *N. gonorrhoeae* from 87.7 to 106.1 million new cases between years 2005 to 2008 (3). While there are no comprehensive data on incidence of *N. gonorrhoeae* from Iran, WHO reports in 2008 indicated 3.1 million new cases of *N. gonorrhoeae* from Eastern-Mediterranean region including Iran (3). Although gonorrhea infections can usually appear asymptomatic, if infection left untreated, it can cause severe complications such as urethritis and cervicitis (2). Syphilis is one of the most important STDs, caused by the bacterium *Treponema pallidum *(4).

The same transmission route and risk factors are possible occurrence of HIV and *T. pallidum* co-infection (5). Despite decades of clinical experience with co-infected patients, the interaction between HIV and syphilis remains complicated and the clinical treatment of co-infected patients remains challenging. (6). There is strong evidence that STDs, especially gonorrhea and syphilis, impact the dynamics of human immunodeficiency virus (HIV) infection (7). These infections facilitate the acquisition of HIV infection via damage to the genital tract or skin (7). Furthermore, gonorrhea and syphilis potentially increase the risk of viral shedding and subsequently transmission of HIV (6, 8).

Recent estimates have suggested that nearly a hundred thousand patients with HIV are living in Iran (9); however, there is no record on the prevalence of *N. gonorrhoeae* co-infection among HIV patients in Iran. The emergence of resistant strains of *N. gonorrhoeae* to wide range of antimicrobial agents is a global challenge to deal with gonorrhea infections (10). Early diagnosis has an important role in reducing unwanted outcomes of gonorrhea and syphilis infections. Therefore, the aim of present study was to determine the frequency of gonorrhea and syphilis co-infection among HIV patients and the potential impact of other risk factors on this situation in the south-west of Iran.

## Methods

**Study setting and design:** This retrospective study was performed within a ten year period from 2004 to 2013 among HIV positive patients at Shiraz HIV/AIDS Research Center. Shiraz HIV/AIDS Research Center as the second HIV/AIDS Research Center in Iran is affiliated to Shiraz University of Medical Sciences, located in Shiraz, the south-west of Iran.  Shiraz HIV/AIDS Research Center specializes in counseling, treatment and research related to HIV/AIDS. 

Totally, 806 HIV patients with complete medical records were enrolled in presents study. Cases without or missing medical records were excluded. This study was approved by the Ethics Committee of Shiraz University of Medical Sciences (Local register code: IR.SUMS.MED.REC. 1395. s11) and was in accordance with the declaration of Helsinki. The ethics committee waived the need for informed consent because we only used medical records and ensured about the patient confidentiality with no personal data. 

**Diagnosis of HIV and** **co-** **infections:** Enzyme linked immunosorbent assay (ELISA) (Dia.Pro Diagnostic Bioprobes, Italy) technique was used for preliminary detection of HIV infection. Subsequently, primary positive results were then confirmed by a Western Blotting test. Syphilis was routinely diagnosed by the consistency of the clinical manifestations and the rapid plasma reagin (RPR) (Bionik, Tehran, Iran) test was used to serological detection.

Gonorrhea was routinely diagnosed by standard microbiological procedures. The specimen was collected based on main criteria for males and females and was identified by using direct gram staining and culturing on selective agar (11). Demographic and clinical data, such as age, gender, prison history, addiction history, transmission route and CD4+ lymphocyte count were also evaluated for all participants. 

**Statistical analysis:** Analysis was performed using SPSS^TM^ software, Version 21.0 (IBM Corp., USA). The results are presented as descriptive statistics in terms of relative frequency. Values were expressed as the mean ± standard deviation (continuous variables) or percentages of the group (categorical variables). Chi–square or Fisher's exact tests was used to estimate any statistical association for quantitative variables, and paired *t*-tests were used to compare means. A p<0.05 was regarded as significant relevance. Logistic regression modeling was performed to identify the factors associated with risk of gonococcal infection among HIV patients. The associations which were presented as odds ratio (OR) together with 95% confidence interval (CI) were considered as significant if the corresponding 95% CI does not include one.

## Results

Of the total 806 HIV patients who were tested for gonococcal infection, 39 (2.6%) cases had gonorrhea. Despite the higher occurrence of gonorrhea infection among older patients, compared to mono-infected ones, no significant differences were found. Compared with mono-HIV infected patients, gonorrhea infection was more likely among males (92.3 vs. 69.4%, P<0.05). Addiction and prison history seems to be a risk factor for gonorrhea infection, since the majority of gonorrhea infected patients, compared to non-infected patients, experienced drug addiction and prison (89.7 vs. 68.4% and 84.6 vs. 60.6%, respectively, P<0.05). Also, the majority of gonorrhea infected patients experienced sexual contact in their life; compared to HIV mono-infected patients, the proportions were significantly higher (79.5 vs. 42.2%, P<0.001). The common HIV infection transmission routes in both gonorrhea infected and non-infected patients was intravenous drug use (IDU) and sexual contact, but patients infected via IDU had a significant risk for co-infection with gonorrhea (P<0.05). 

Among the clinical risk factors evaluated, there was no significant association between highly active antiretroviral therapy (HAART) and gonorrhea infection. Also, although compared with HIV mono-infected, the mean of CD4+lymphocyte was higher among gonorrhea infected patients (368±238 vs. 415±328, respectively), but the difference was not significant. The full results of demographic and clinical characteristics of HIV and gonorrhea infection among the studied cases was shown in Table 1. Logistic regression analysis showed that sexual contact significantly increased the chances of gonococcal infection about 4 fold (OR: 4, CI: 1.7-9.39, P=0.001). The full results of logistic regression modeling for risk factors associated with gonococcal co-infection are shown in table 2. Based on the laboratory results, of the totally tested HIV patients for syphilis infection, no positive case was found by RPR test.

**Table 1:** Demographic and clinical characteristics of HIV and gonorrhea infection among the studied cases

**Groups** **Studied factors**	**HIV ** **mono-infected** **n (%)** **(Total=767)**	**Gonorrhea co-infected** **n (%)** **(Total=39)**	**p-value**
**Age (years)**			
Mean ± SD	38±9	40±8	0.17
range	2-70	24-61	
<19 years old	20 (2.6)	0	
20-50	677 (88.3)	35 (89.7)	0.36
>51	70 (9.1)	4 (10.3)	
**Gender**			
Male	532 (69.4)	36 (92.3)	0.002
Female	235 (30.6)	3 (7.7)	
**HAART ** ***therapy***			
Yes	517 (67.4)	25 (64.1)	0.66
No	250 (32.6)	14 (35.9)	
**Addict history**			
Yes	525 (68.4)	35 (89.7)	0.005
No	242 (31.6)	4 (10.3)	
**Prison history**			
Yes	465 (60.6)	33 (84.6)	0.003
No	302 (39.4)	6 (15.4)	
**Blood transfusion history**			
Yes	49 (6.4)	2 (5.1)	0.75
No	718 (93.6)	37 (94.9)	
**Sexual ** **contact**			
Yes	324 (42.2)	31 (79.5)	0.001
No	443 (57.8)	8 (20.5)	
**Transmission route**			0.006
Intravenous drug use	448 (58.4)	33 (84.6)	
**Sexual contact**	250 (32.6)	4 (10.3)	
**Mother to Infant**	18 (2.4)	0	
Blood transfusion	3 (0.4)	1 (2.5)	
Occupational exposure	1 (0.1)	0	
Unknown	47 (6.1)	1 (2.5)	
**CD4+lymphocyte**			
Mean ± SD	368±238	415±328	0.23
Range	10-1678	21-1691	
<200/μl Cells/mm^3^	182 (23.7)	7 (17.9)	0.56
≥200/μl Cells/mm^3^	585 (76.3)	32 (82.1)	

**Table 2:** Logistic regression modeling for factors associated with risk of gonococcal infection among HIV patients

**Factor**	**Subgroup**	**OR**	**(95% CI)**	**Significant level**
Age	Age	1.03	(0.99-1.07)	0.13
Gender	Female	Ref		0.31
	Male	2.51	(0.42-15.07)	
HAART therapy	No	Ref		0.99
	Yes	1	(0.49-2.03)	
Addict history	No	Ref		0.30
	Yes	0.3	(0.03-2.96)	
Prison history	No	Ref		0.48
	Yes	1.69	(0.4-7.15)	
Blood transfusion history	No	Ref		0.48
	Yes	0.59	(0.14-2.58)	
Sexual contact	No	Ref		0.001
	Yes	4	(1.7-9.39)	
Transmission route	Other	Ref		0.25
	Intravenous drug use	2.37	(0.54-10.42)	
CD4+lymphocyte	<200/μl Cells/mm^3^	Ref		0.27
	≥200/μl Cells/mm^3^	1.63	(0.69-3.85)	

## Discussion

STDs include a range of infections that, regardless of gender, usually lead to a clinical complication (2). Gonorrhea, as an important cause of STDs, can lead to a syndemic relationship with HIV/AIDS (12). Physical and molecular role of gonorrhea infection in facilitating HIV acquisition and transmission is undeniable; besides the risk of resistance, gonorrhea could become a serious concern of public health (7, 10). Control and prevention of this co-infection need an adequate surveillance in every part of the world (7, 13). Given this fact, our results revealed the prevalence of gonorrhea infection 2.6% among HIV patients in Shiraz, southwest of Iran. To the best of our knowledge, this is the first report from Iran and there is no previous record on the prevalence of HIV/gonorrhea co-infection to compare of our situation with others in neighborhood regions. Nonetheless, compared to previous reports on the prevalence of gonorrhea infection among non- HIV infected individuals from different parts of our country, gonorrhea infection among HIV patients seems to have a relatively higher rate; yet, most of these studies are conducted among women (2, 14-16). Similar reports from other parts of the world indicated the variability of gonorrhea infection among HIV patients (8, 17-19), which could originate from diagnostic or lifestyle differences in these regions (14). However, the prevalence reported in our study (2.6%) is lower than those reported among HIV positive patients in Kenya (17%) (20), capital of India (9%) (21), and Arizona state of the USA (4.5%) (12), whereas it is higher than those reported from California state of USA (2%), India (2%) , and Brazil (0%) (18). Furthermore, besides the higher incidence of gonorrhea infection in men, among the other socio-demographic factors that were studied, history of drug addiction, prison and sexual contact is a significant risk factor for occurrence of gonorrhea co-infection. The relationship of these risk factors has also been reported in other studies (22). Although occasion of gonorrhea acquisition in our studied patients are unavailable (inside or outside the prison), one of the reasons for higher chance of gonorrhea infection among male prisoners attributed to more tendency for risky behaviors, such as unprotected intercourse and also lower knowledge about STDs (23, 24). Rectal gonorrhea and anal warts were mentioned as important risk factors for HIV acquisition (25).

In our study, assessment of CD4+ lymphocyte count revealed a higher mean of CD4+ lymphocyte among gonorrhea infected than non-infected patients, although the difference was not statistically significant, Levine* et al.* previously showed that the median of endocervical CD4+ lymphocyte was significantly greater among patients with STDs than among those without them and it may facilitate HIV transmission by increasing the presence of CD4+ lymphocytes at endocervix (26). Hopefully, in our results none of the HIV patients had syphilis co-infection. The only data on prevalence of *T. pallidum *among Iranian HIV-positive patients by Badie et al. showed 0.45% co-infection (5). Other attempts on the determination of syphilis prevalence among Iranian high risk groups showed low rates of detection closest to our findings (27-29).Self-medication or antibiotic therapy for treating irrelevant diseases may be one of the main causes of such observations. 

Finally, our study had some limitations. Although the traditional approaches, such as the use of selective culture, is still the preferred laboratory test for diagnosis of gonorrhea, for some conditions and specimens, the nucleic acid amplification tests (NAATs) have a higher sensitivity compared to traditional methods (1). Moreover, it would be better to have access to time of gonorrhea detection (before or after HIV infection) for determining the probable effect of using HAART on the prevalence of HIV/gonorrhea co-infection. 

In conclusion, despite the limitations, our study, as a preliminary survey, provided unique information on the prevalence of gonorrhea and syphilis co-infections among HIV infected patients for clarification of our situation and also comparison with others. Additionally, we introduced the main risk factors associated with gonorrhea co-infection in HIV patients in our region which could provide a good background for effective management of prevalence and prevention of these diseases.
